# TIL-Derived CAR T Cells Improve Immune Cell Infiltration and Survival in the Treatment of CD19-Humanized Mouse Colorectal Cancer

**DOI:** 10.3390/cancers15235567

**Published:** 2023-11-24

**Authors:** Can Zhu, Yuanyuan Zhao, Jiaheng He, Huan Zhao, Li Ni, Xinyi Cheng, Yida Chen, Liqian Mu, Xiaojun Zhou, Qin Shi, Jie Sun

**Affiliations:** 1 Department of Orthopedics, The First Affiliated Hospital of Soochow University, Orthopedic Institute of Soochow University, Suzhou Medical College, Soochow University, 899 Pinghai Road, Suzhou 215031, China; 20224032012@stu.suda.edu.cn (C.Z.); zhaohuan@suda.edu.cn (H.Z.); nili@suda.edu.cn (L.N.); 20224232047@stu.suda.edu.cn (X.C.); 20224232049@stu.suda.edu.cn (Y.C.); 2 National Clinical Research Center for Hematologic Diseases, The First Affiliated Hospital of Soochow University, 899 Pinghai Road, Suzhou 215031, China; zyy@suda.edu.cn; 3 Department of Pathology, School of Biology and Basic Medical Sciences, Suzhou Medical College, Soochow University, Suzhou 215123, China; 4 Department of Orthopaedics, The Affiliated Jiangsu Shengze Hospital of Nanjing Medical University, No. 1399, Market West Road, Shengze Town, Suzhou 215000, China; 18896719637@163.com; 5 Department of General Surgery, The First Affiliated Hospital of Soochow University, Suzhou Medical College, Soochow University, 899 Pinghai Road, Suzhou 215031, China; muliqian@suda.edu.cn (L.M.); chowxj@126.com (X.Z.)

**Keywords:** immunotherapy, tumor-infiltrating T cell, CAR T, colorectal cancer

## Abstract

**Simple Summary:**

Lack of a tumor-specific antigen as the target and an inhospitable tumor environment limit the clinical application of Chimeric antigen receptor-engineered T cells (CAR Ts) in solid tumors, compared with its unprecedented results in treating hematological cancers, while tumor-infiltrating T lymphocytes (TIL) exhibit diverse T cell receptor clonality and superior tumor-homing abilities. In our study, we took advantage of the dual advantages of TIL-T and CAR T and constructed TIL CAR-T cells capable of MHC molecules and co-stimulatory signal-independent activation with tumor-specific broad-spectrum TCR expression. We innovatively constructed a humanized mouse colorectal cancer model, which provides an important platform for subsequent evaluation of human CD19-target CAR T cells. Meanwhile, TIL CAR-T cells have better anti-tumor efficacy than conventional CAR T, characterized by a better ability to enhance tumor invasion, relieve T cell exhaustion, and promote immune memory.

**Abstract:**

Chimeric antigen receptor-engineered T cells (CAR Ts) targeting CD19 have shown unprecedented prognosis in treating hematological cancers. However, the lack of a tumor-specific antigen as the target and an inhospitable tumor environment limit the clinical application of CAR T in solid tumors. Tumor-infiltrating T lymphocytes (TIL) exhibit diverse T cell receptor clonality and superior tumor-homing abilities. Therefore, in our study, human CD19-target TIL CAR-Ts armed with CD3ζ and 4-1BB signaling domains were constructed. Mouse colorectal cancer CT26 cells expressing human CD19 (hCD19^+^-CT26) were developed to assess the anti-tumor activity of TIL CAR-T cells, both in vitro and in vivo. Compared with splenic CAR T adoptive transfer, TIL CAR-T administration showed superior tumor suppression ability in hCD19^+^-CT26 tumor-bearing mice. Furthermore, more T cells were found at the tumor site and had lower exhaustion-related inhibitory receptor (T cell immunoglobulin and mucin domain-containing protein 3, Tim3) expression and higher immune memory molecule (CD62L) expression. Overall, we provided an artificial tumor-specific antigen in solid tumors and demonstrated that combined CAR-expressing TIL-Ts (TIL CAR-Ts) exhibited strong anti-tumor activity, with improved T cell infiltration and immune memory. Our humanized tumor antigen presented platform of mice suggests that TIL CAR-T-based adoptive therapy could be a promising strategy for solid cancer treatment.

## 1. Introduction

Chimeric antigen receptor-engineered T cells (CAR Ts) are emerging as a promising novel technology for cancer immunotherapy [1]. The structure of CAR-modified T cells consists of an extracellular antigen-recognition domain, antibody single-chain antibody fragments (scFvs), which recognize and bind the cell surface antigen independent of the major histocompatibility complex (MHC) molecule, a hinge/transmembrane domain (e.g., the homodimer of CD3 or CD8), and an intracellular domain that provides activation signals to T cells (CD3ζ and co-stimulatory signaling molecules, e.g., 4-1BB, CD28, or OX40) [2]. CAR-modified T cells specifically recognize antigens expressed on tumor cell surfaces, whereby activating intracellular signaling and subsequently inducing cytotoxic activity, which is crucial for eliminating tumor tissues.

In the past ten years, CAR T technology has caused a significant revolution in the treatment of hematological malignancies. CAR Ts targeting CD19, including CD3ζ and CD28 or 4-1BB co-stimulatory signaling domains, have shown remarkable success in treating B-cell malignancies [3]. The complete remission rates (CRR) of patients with relapsed or refractory B-cell malignancies achieved up to 90% [4]. Since the Food and Drug Administration approved CD19 CAR T cells for treating B-cell malignancies [5], numerous attempts have been made to mimic this strategy for solid tumors, which comprise the vast majority of cancers. In contrast, most clinical studies on CAR T for solid tumors have limited efficacy, with less than 25% response rates [6,7]. Compared with hematological malignancies, CAR T therapy for solid tumors presents unique challenges, and several hurdles limit its efficacy [8].

Target antigen selection is a major determinant of the efficacy and safety of CAR T therapy for solid tumors. CD19 provides a near-perfect target for B-cell malignancies, as it is only expressed in hematopoietic B cells. Although the CD19-targeting CAR induces B-cell aplasia, it is clinically manageable and not fatal. Due to the paucity of tumor-specific antigens (TSAs), tumor-associated antigens (TAAs) are selected as the CAR Ts’ target molecules. Consequently, healthy cells that share TAAs are innocently killed, while tumor cells can escape clearance by dynamically regulating the expression of target antigens, resulting in “on-target, off-tumor” toxicities.

Moreover, the heterogeneity of solid tumor antigens enables them to escape CAR T surveillance, which usually recognizes only a single antigenic target that is not expressed across all tumor cells and cannot eradicate all malignant cells. For example, a clinical study on EGFRvIII-targeted CAR Ts in glioblastoma showed that relapse still occurred even if all EGFRvIII-positive tumor cells were eliminated [9,10]. However, expanding CAR T targeting will increase the risk of “on-target, off-tumor” toxicities because the selected target antigens are TAAs rather than TSAs. Another major hurdle is the infiltration and survival of CAR T cells in solid tumors. Contrary to dispersive and circulating hematological tumor cells, solid tumor cells are usually surrounded by physical barriers that prevent CAR T cell infiltration and survival. With this powerful technology, continuous efforts are required to improve the specificity and broad-spectrum targeting of CAR T cells and augment the infiltration, survival, and persistence of solid tumors.

Accumulating evidence suggests that endogenous T cells can be primed through the recognition of tumor neoantigens and infiltration into solid tumor tissue. The success of immune checkpoint inhibitor (ICI) therapy has demonstrated the presence of tumor-primed and -infiltrating lymphocytes (TIL-Ts) [11]. There are several congenital advantages to TIL-Ts over circulating T cells. TIL-Ts have intrinsic properties of homing and infiltration to tumor sites, which is a prerequisite for T cell surveillance. TIL-Ts contain multifarious and broad-spectrum neoantigen-reactive T cell receptors (TCRs) that can distinguish malignant cells from healthy cells and specifically target heterogeneous antigens conducive to overcoming antigen escape and ameliorating “on-target, off-tumor” toxicity [12,13]. Thus, the plasticity and potential of TIL-Ts have broad prospects in anti-tumor therapy.

These are the inherent limitations of TIL for tumor immunotherapy, as evidenced by only a subset of patients showing a durable response to adoptive TIL-T therapy [14]. Tumor cells have numerous mechanisms to escape TIL-T surveillance, the most important of which is the downregulation or lack of MHC and co-stimulatory molecules on the tumor cell surface, which produces TIL-Ts in a state of anergy [15,16]. Moreover, most TIL-Ts exhibit exhausted phenotypes [17]. Considering the advantages of TIL-Ts and CAR Ts, we hypothesized that their rational combination could overcome their respective shortcomings and become an alternative T cell-based anti-tumor strategy.

In this study, we genetically engineered mouse colorectal cancer cells (CT26) with a human CD19 molecule (hCD19^+^-CT26) to provide a TSA for human CD19-targeting CAR Ts. This can be achieved in vivo by oncolytic viruses (OV). Our results showed that the expression of hCD19 did not affect the tumorigenicity of CT26 cells in immunologically sound BALB/c mice. These hCD19^+^-CT26 cells can be recognized and killed by hCD19-targeting CAR T cells that contain CD3ζ and 4-1BB signaling molecules (CD19-BBζ CAR). Subsequently, CD19-BBζ CAR-engineered TIL-T cells (TIL CAR-Ts) were developed. The introduction of CD19-BBζ CAR into TIL-Ts increases persistence and delays exhaustion. In the CD19-humanized colorectal cancer-bearing mouse model, TIL CAR-Ts penetrated the tumor site, decreased the tumor burden, and prolonged survival without any observed “off-target” toxicity. Splenic T cells of mice administered TIL CAR-T upregulated pro-inflammatory cytokine expression and downregulated T cell immunoglobulin and mucin domain-containing protein 3 (Tim3) expression, resulting in immune memory. In conclusion, CAR-armed TIL-Ts improve T cell infiltration and survival, forming a long-term memory pool for immune surveillance. This may be a new strategy to improve the effectiveness of conventional CAR T cells against solid tumors.

## 2. Materials and Methods

Female BALB/c mice (6 to 8 weeks old) were purchased from Charles River (Beijing, China) and kept in a constant temperature and humidity-specific pathogen-free (SPF) environment. All animal procedures were approved by the Animal Care and Use Committee of Soochow University. All cell lines involved in this study were purchased from American Type Culture Collection (ATCC, Manassas, VA, USA).

### 2.1. Establishment of Human CD19-Expressed Tumor Cell Line

The sequence of human *cd19* was acquired from the Raji cell line by PCR and introduced into the murine stem cell virus-zeocin-resistant vector (MSCV-zeocin^+^) by genetic engineering (MSCV-hCD19). The MSCV-hCD19 retrovirus was packaged in Platinum-E (Plat-E) cells, and the colorectal cancer CT26 and osteosarcoma K7M2 cell lines were infected. The cells were stained with allophycocyanin (APC)-conjugated anti-human CD19 antibodies (BioLegend, 302211, San Diego, CA, USA). The expression of human CD19 on CT26 (hCD19^+^-CT26) and K7M2 (hCD19^+^-K7M2) cells was detected by FACS (BD, Franklin Lakes, NJ, USA). The hCD19^+^-CT26 and hCD19^+^-K7M2 cells were subjected to FACS and expanded for further use after the expression of human CD19 exceeded 90%. Meanwhile, CT26 and K7M2 cells infected with the MSCV-zeocin^+^ retrovirus were designated as mock-CT26 and mock-K7M2 cells, respectively.

### 2.2. Construction of CD19-BBζ CAR Expression Plasmid and Retrovirus

The encoding sequence of *cd19-bbζ* CAR consists of a signal peptide of mouse CD8α, hCD19-targeting single-chain variable fragments (scFvs), truncated hinge and transmembrane domains of mouse CD8α, and cytoplasmic regions of mouse 4-1BB (CD137) and CD3ζ. The sequence of scFvs is derived from the hybridoma cell line FMC63 [18]. The *cd19-bbζ* CAR sequence was fused with *gfp* sequences using T2A and cloned into the plasmid pMSCV.

Gene segments were synthesized by General Biotechnology Co., Ltd. (Chuzhou, Anhui, China) and sequenced by Genewiz Biotechnology Co., Ltd. (Suzhou, Jiangsu, China). Retroviruses were produced by transient transfection of Plat-E packaging cells with viral plasmids and retroviral packaging plasmids pCL-Eco, using the calcium phosphate method. The virus was collected and concentrated 48 h after transfection. The concentrated virus infected NIH/3T3 cells (5000 cells/well) in a gradient, and FACS detected GFP expression after 72 h. The titer was calculated with the following formula: virus titer (viruses/mL) = (5000 × positive rate (%) × dilution)/Volume added (mL).

### 2.3. Activation of Splenic T Cells from the BALB/c Mice

Pan CD3^+^ T cells were isolated from the splenic cells of 6-week-old female BALB/c mice using magnetic beads (Miltenyi Biotec, 130-095-130, Bergisch Gladbach, Germany) according to the manufacturer’s protocol and cultured in anti-mouse CD3 and CD28 (BioLegend, 100340, 102116) antibody-coated 24-well plates at a density of 2–3 million/well in Roswell Park Memorial Institute 1640 medium (Hyclone, Logan, UT, USA) containing 10% fetal bovine serum (Gibco, Billings, MT, USA), 10 ng/mL IL-2 (Peprotech, Cranbury, NJ, USA), and 50 μM 2-mercaptoethanol for 24–36 h.

### 2.4. Construction of CD19-BBζ CAR T Cells (CAR T)

After T cell activation, T cells were infected twice with the corresponding virus according to MOI (virus/cell) = 3 and the infection-promoting agent protamine (10 µg/mL, Sigma, St. Louis, MO, USA) at 24 h intervals. T cells expressing CD19-BBζ CAR were named CD19-BBζ CAR T cells and were infected with the MSCV-GFP retrovirus as mock T cells. At 48 h after infection, the percentage of CAR-positive cells was determined and sorted by FACS.

### 2.5. Establishment of Tumor-Bearing Mice

Six-week-old female BALB/c mice were subcutaneously inoculated with hCD19^+^-CT26 cells (5 × 10^5^/mouse). Tumor growth was monitored periodically by a Vernier caliper. Tumor volume was calculated with the formula: volume = (Width × Length^2^)/2.

### 2.6. Preparation of TIL CD19-BBζ CAR-T Cells (TIL CAR-T)

When the tumor size reached approximately 108 mm^3^, the mice were euthanized, and the tumor tissues were collected aseptically. Mononuclear cells from the tumor tissues were prepared as previously described [19]. TIL-Ts were acquired by magnetic bead sorting in the same manner as splenic T cells. TIL-Ts were infected with MSCV-GFP mock and MSCV-CD19-BBζ CAR retroviruses and named mock TIL-T and TIL CAR-T, respectively. The mock TIL-T and TIL CAR-T cells were further purified by FACS.

### 2.7. Assessment of CD19-BBζ CAR T Cytotoxicity

TIL CAR-T cells/CAR T cells were co-cultured with the hCD19^+^-CT26 cells in round-bottom 96-well plates at different ratios of T cells to target tumor cells. After 48 h, the cell morphology was observed and photographed with an inverted microscope (Zeiss, Oberkochen, Germany). To detect the effector phenotype of T cells, GolgiPlug protein transport inhibitor (BD, USA) was added to the co-culture system and cultured for 4 h. The supernatant T cells were harvested and incubated with PerCP/Cyanine5.5-conjugated anti-mouse CD8a (BioLegend, 100733), APC-conjugated anti-mouse TNFα (BioLegend, 506307), and PE-conjugated anti-mouse IFNγ (BioLegend, 505807) antibodies. The expression of IFNγ and TNFα in CD8^+^ T cells was assayed by FACS.

After T cell removal, 200 μL of complete medium containing a 10% resazurin (Sigma-Aldrich, TOX8) was added to each well and incubated for 3 h. After shaking gently for 5 min on a shaker in the dark, the optical density (OD) of the supernatant was measured using a full-wavelength spectrophotometer (BioTek, Winooski, VT, USA) at 600 nm. The fluorescence intensity was detected at an excitation wavelength of 560 nm and an emission wavelength of 590 nm.

### 2.8. Adoptive Cell Therapy (ACT)

Seven days after tumor inoculation, the mice were randomly divided into five groups. They received different cell transfusions through the tail vein. (1) phosphate buffer saline (PBS) only, PBS group; (2) MSCV-GFP retrovirus-infected splenic T cells, mock T group; (3) splenic CD19-BBζ CAR T cells, CAR T group; (4) tumor-derived MSCV-GFP retrovirus-infected T cells, mock TIL-T group; and (5) tumor-derived CD19-BBζ CAR T cells, TIL CAR-T group. Endogenous lymphocytes were depleted by intraperitoneal injection of cyclosporine A (100 mg/kg; APE BIO, Houston, TX, USA) 48 h before cell transfusion. Seven days after adoptive cell therapy, tumor-bearing mice were euthanized. Spleens and peripheral blood were collected for T cell phenotype analysis by FACS. In addition, tissues, including tumors, hearts, lungs, and kidneys, were collected for histological analysis.

### 2.9. Flow Cytometry Analysis

Peripheral blood was collected, erythrocytes were lysed with erythrocyte lysis solution, and the remaining cells were stained with APC-conjugated anti-mouse CD3 (BioLegend, 100236) and detected by FACS. Splenocytes were prepared and incubated with APC-conjugated anti-mouse CD3, Brilliant Violet 421-conjugated anti-mouse CD4 (BioLegend, 100437), PerCP/Cyanine5.5-conjugated anti-mouse CD8a, PE/Cyanine7-conjugated anti-mouse CD62L (BioLegend, 104417) and PE/Cyanine7-conjugated anti-mouse Tim3 (BioLegend, 134009) antibodies. In addition, a few cells were stimulated with Cell Activation Cocktail (BioLegend, USA) for 4 h and then stained with APC-conjugated anti-mouse TNFα and PE-conjugated anti-mouse IFNγ antibodies.

### 2.10. Histological Analysis

Tumor tissues were embedded in the Tissue-Tek optimal cutting temperature (O.C.T) compound (Sakura Finetek, Tokyo, Japan). After freezing, they were sliced into 6 μm thin sections and stored at −20 °C. Other tissues, such as the heart and liver, were fixed with 4% paraformaldehyde, dehydrated with a sucrose gradient, and embedded with O.C.T compound for subsequent sections. The sections were warmed to room temperature and then rinsed with H&E (Solarbio, Beijing, China) for a few seconds. After dehydration with gradient alcohol, the sections were left in xylene for 5 min and sealed with neutral resin (Sigma, USA).

The FITC-conjugated IL-11Rα-targeting peptide was synthesized by QYAOBIO (Shanghai, China), and its recognition of CT26 cells was detected by flow cytometry at the concentration of 1 μg/μL. For immunofluorescence staining, tumor tissue sections were incubated with FITC-conjugated IL-11Rα-targeting peptide, DAPI (Sigma) and Alexa Fluor^®^ 647-conjugated anti-mouse CD4 (BioLegend, 100533) or Alexa Fluor^®^ 647-conjugated anti-mouse CD8a (BioLegend, 100727), and DAPI (Yuanye Bio-Technology, Shanghai, China). The TdT-mediated dUTP nick-end labeling (TUNEL) apoptosis assay was performed according to the manufacturer’s instructions (Beyotime, Haimen, Jiangsu, China). The samples were imaged using a fluorescence microscope (Zeiss, Germany).

### 2.11. Statistical Analysis

All statistical analyses were performed using the GraphPad Prism software (version 8.0). Data are shown as mean ± SD. For experiments with one variable, two-sided Student’s unpaired t-tests were performed. A two-way analysis of variance (ANOVA) (unpaired samples) or a two-way repeated measures ANOVA (paired samples) was used to determine significance when two variables appeared. *p* < 0.05 were considered statistically significant (* p < 0.05; ** p < 0.01; *** p < 0.001).

## 3. Results

### 3.1. Infected Ratio of MSCV-CD19-BBζ CAR Retrovirus

**Figure 1:**
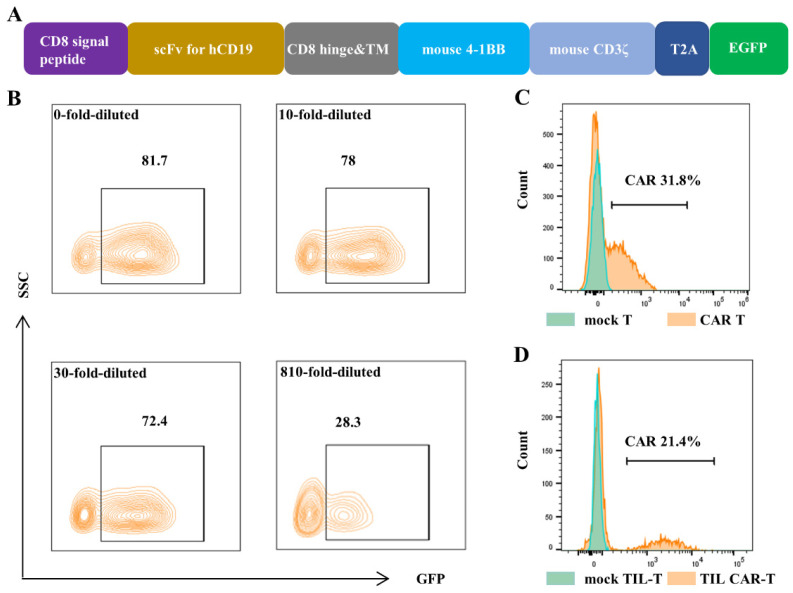
Construction of CAR T cells. The MSCV-CD19-BBζ CAR retrovirus was packed, and then it infected NIH/3T3 cells, splenic T cells, and TIL-Ts separately. The expression of GFP and CAR was detected by FACS. (**A**) Gene structure diagram of *cd19-bbζ car-egfp*. (**B**) The expression of GFP in NIH/3T3 cells, which were infected with different dilution concentrations of virus supernatant. (**C**) The expression of CAR in the splenic T cells after virus infection. (**D**) The expression of CAR in the tumor-derived T cells (TIL-Ts) after virus infection.

The encoding sequence of *cd19-bbζ CAR* was composed of a signal peptide of mouse CD8α, hCD19-targeting single-chain variable fragments (scFvs), truncated hinge and transmembrane domains of mouse CD8α, and cytoplasmic regions of mouse 4-1BB (CD137) and CD3ζ (Figure 1A). The sequence of cd19-bbζ CAR was confirmed using sequencing analysis. The plasmid MSCV-CD19-BBζ CAR or MSCV-GFP was co-transfected with retroviral packaging plasmids in Platinum-E (Plat-E) cells. The viruses were concentrated and used to infect NIH/3T3 cells. The green fluorescence protein (GFP) expression of infected NIH/3T3 cells, detected using fluorescence-activated cell sorting (FACS), was 82.03 ± 0.31%. The concentrated virus was diluted 10 times, and the GFP expression was 79.8 ± 1.59%. For the sample diluted 810-fold, the GFP expression was 30.07 ± 1.70% (Figure 1B). Based on the diluted fold and the GFP expression, the titer of the virus concentrate in the infected cells was calculated as 1.22 ± 0.069 × 10^8^/mL.

Splenic T cells and tumor-derived TIL-Ts were infected with a multiplicity of infection = 3 (MOI = 3) twice at an interval of 24 h. Fluorescence-activated cell sorting (FACS) results showed that the CAR expression of CAR T and TIL CAR-T was 32.3 ± 0.68% and 20.77 ± 0.65% 3 d after infection, respectively (Figure 1C,D). 

### 3.2. Construction of hCD19-Expressed Tumor Cells

CD19 is rarely expressed in solid tumors. To test the efficacy of CD19 CAR T cells, mouse colorectal cancer CT26 and osteosarcoma K7M2 cells were genetically engineered to express human CD19 transmembrane molecules. FACS detected human CD19 expression in mock-CT26, mock-K7M2, hCD19^+^-CT26, and hCD19^+^-K7M2. After FACS was completed, CD19 expression on hCD19^+^-CT26 and hCD19^+^-K7M2 was 94.57 ± 0.6% and 96.4 ± 0.6%, respectively, for further use (Figure S1).

### 3.3. Effect of TIL CAR-T Cells on hCD19-Humanized CT26

**Figure 2:**
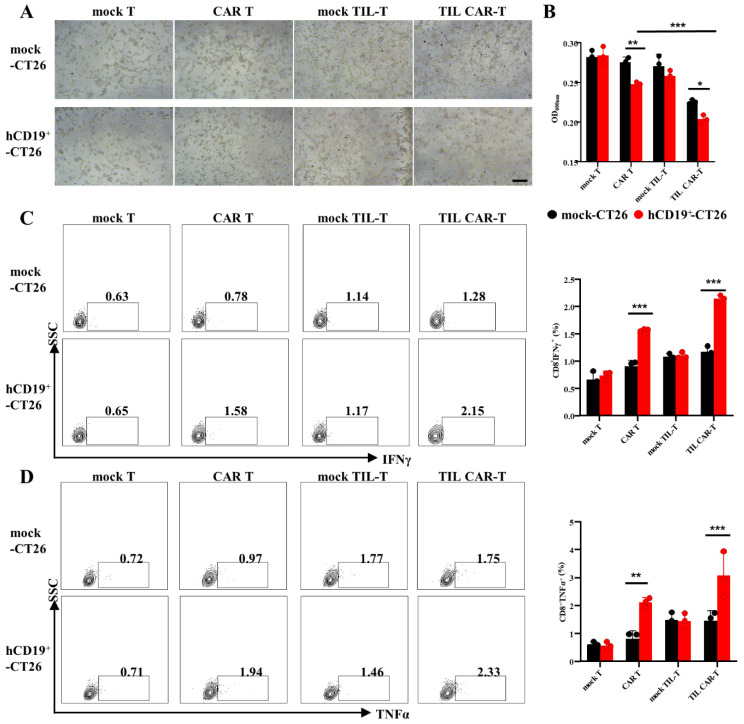
The killing efficiency of CD19-BBζ CAR T cells on hCD19-humanized CT26. (**A**) Representative images of T cells co-cultured with CT26 (scale bar = 500 μm). (**B**) OD value of CT26 cells detected by resazurin dye solution. (**C**) The percentage of IFNγ^+^CD8^+^ T cells detected by FACS. (**D**) The percentage of TNFα^+^CD8^+^ T cells detected by FACS (*n* = 3, * *p* < 0.05; ** *p* < 0.01; *** *p* < 0.001).

CD19-BBζ CAR-positive TIL-T cells were sorted using FACS and co-cultured with hCD19^+^-CT26 cells. As shown in Figure 2A, CAR T and TIL CAR-T cells had an obvious killing effect on hCD19^+^-CT26 tumor cells, and the cells lost their original contour and broke into cell debris. Moreover, CAR T and TIL CAR-T showed obvious proliferation and clonal formation. Quantitative analysis of a resazurin dye kit and TUNEL staining showed that TIL CAR-T had stronger killing and pro-apoptotic effects on hCD19^+^-CT26 cells than the CAR T group (Figure 2B and Figure S2). In addition, the cytotoxicity of CAR T cells on another CD19^+^ tumor cell line (hCD19^+^-K7M2) showed a trend similar to that of hCD19^+^-CT26 cells (Figure S3).

T cells play a cytotoxic role partly through the secretion of IFN and TNFα. Therefore, the expression of IFNγ and TNFαs in T cells were detected by FACS. As shown in Figure 2C,D, the expression of IFNγ in CAR T or TIL CAR-T co-cultured with the hCD19^+^-CT26 groups was 1.57 ± 0.02% and 2.14 ± 0.07%, while the expression of TNFα was 2.12 ± 0.17% and 3.09 ± 0.82%, respectively; these expression values were significantly higher than that of the other groups. For mock TIL-T, there was no significant difference in IFNγ and TNFα between mock-CT26 and hCD19^+^-CT26, but the killing ability was slightly stronger than the mock T group. These results demonstrated that both CAR T and TIL CAR-T cells could effectively kill the CD19^+^-targeted cells, but TIL CAR-T performed better, which might be a tonic role of tumor antigen-specific TCR.

### 3.4. TIL CAR-T Suppressed Tumor Growth In Vivo

**Figure 3:**
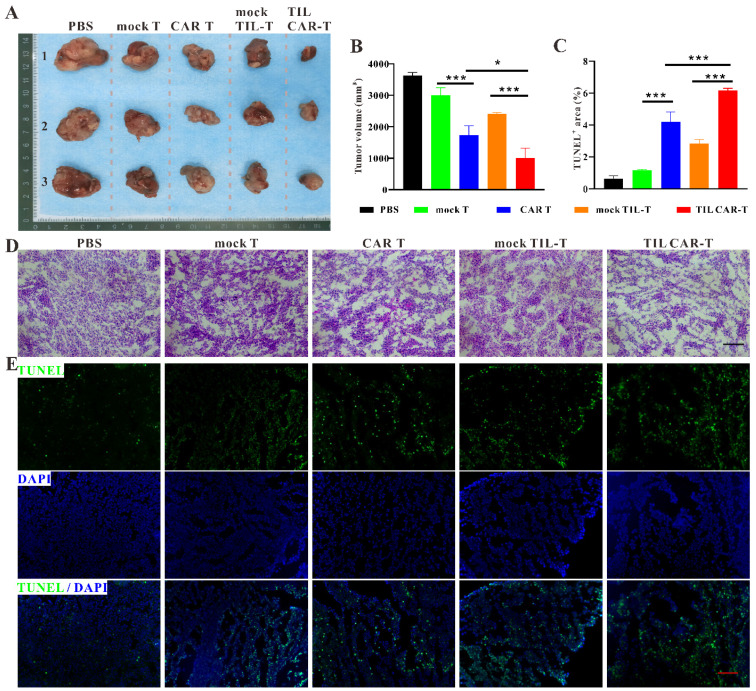
TIL CAR-T inhibited tumor growth. (**A**) Representative gross view of tumor in tumor-bearing mice. (**B**) Statistical graph of tumor size. (**C**) Quantification of TUNEL staining. (**D**) H&E staining of tumor tissues. (**E**) TUNEL (green) and DAPI (blue) fluorescent staining of tumor sections (scale bar = 200 μm, *n* = 3, * *p* < 0.05; *** *p* < 0.001).

To explore the effect of TIL CAR-T cells on tumor progression, TIL CAR-T cells were injected into hCD19^+^-CT26 colorectal cancer-bearing mice via the tail vein. As expected, the tumor volume of all the T cell therapy groups was smaller than that of PBS group. In addition, the tumor volumes of the CAR T and TIL CAR-T cell therapy groups were significantly smaller than those of the corresponding mock T cell therapy group. The tumor size in the TIL CAR-T group was significantly smaller than that in the CAR T group (Figure 3A,B). According to the results of hematoxylin and eosin (H&E) staining, there was no significant difference in the internal structure of tumor tissues between the PBS and mock T groups. However, a difference was observed between the PBS and CAR T, mock TIL-T, and TIL CAR-T groups (Figure 3D). Compared with the mock TIL-T cell group, the TIL CAR-T group showed the looser internal structure of the tumor tissue, manifesting a decrease in tumor cells.

TUNEL staining showed that apoptosis occurred in tumor tissue following T cell injection. However, TIL CAR-T cells induced significant cell apoptosis compared with the other groups (Figure 3C,E). The data showed that TIL CAR-T cells had a specific cytotoxic effect on hCD19^+^-CT26-induced tumors, resulting in increased apoptosis of tumor cells.

### 3.5. TIL CAR-T Promoted Immune Cell Infiltration in the Tumor Tissues

**Figure 4:**
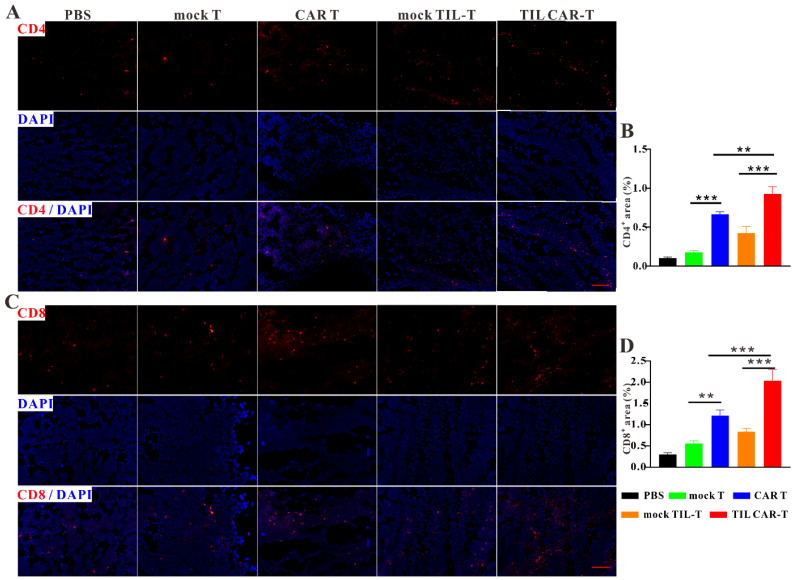
TIL CAR-T promoted immune cell infiltration in the tumor tissues. (**A**) Representative images of CD4 (red) and DAPI (blue) fluorescence staining. (**B**) Quantitative CD4 fluorescence staining area of tumors. (**C**) Representative images of CD8 (red) and DAPI (blue) fluorescence staining. (**D**) Quantitative CD8 fluorescence staining area of tumors (scale bar = 200 μm, *n* = 3, ** *p* < 0.01; *** *p* < 0.001).

We explored immune cell infiltration in tumor tissues. Immunofluorescence staining showed that more CD4^+^ and CD8^+^ T cells were found in tumor tissues with T cell adoption than in the PBS groups, while the frequencies of CD4^+^ and CD8^+^ T cells in the CAR T and TIL CAR-T cell groups were statistically higher than those of their corresponding mock T groups (Figure 4). Many studies had shown that IL11Rα was highly expressed in colorectal cancer cells and was often used as a target for immunotherapy [20,21,22,23]. Our FACS and cellular immunofluorescence staining results also showed that IL-11Rα was highly expressed on the surface of CT26 cells. (Figure S4A,B). Therefore, we performed immunofluorescence staining with FITC-conjugated IL-11Rα-targeting peptides in combination with anti-CD4 or anti-CD8 antibodies, respectively, to reflect the co-localization of tumor-infiltrating T cells and tumor cells in the tumor tissues. The results showed that tumor-bearing mice treated with TIL CAR-T cells had more infiltration of CD4^+^ and CD8^+^ T cells than those in the other groups (Figure S4C,D). It provided the antecedent condition for immune cell infiltration into the tumor and facilitated the killing of tumor cells.

### 3.6. TIL CAR-T Enhanced the Cytotoxicity of Splenic T Cells

**Figure 5:**
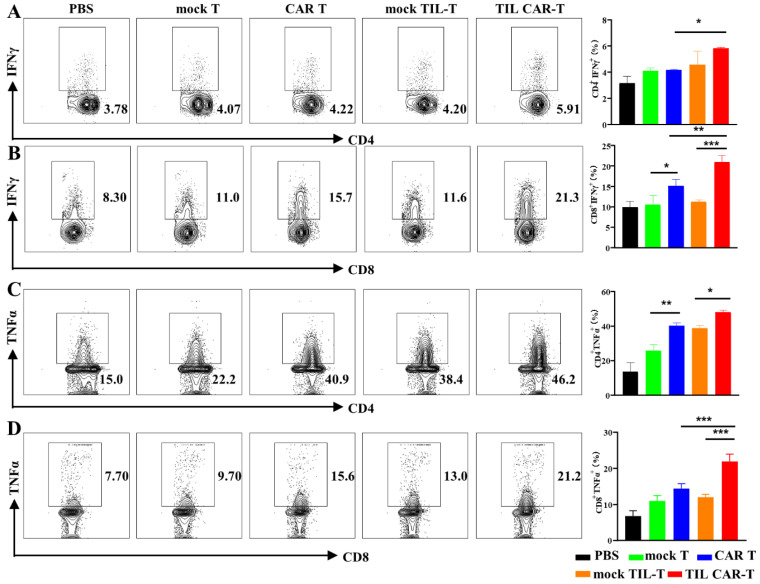
TIL CAR-T enhanced the cytotoxicity of splenic T cells of the tumor-bearing mice. The frequency of subtype T cells in the spleen was detected by FACS. (**A**) The percentage of IFNγ^+^CD4^+^ T. (**B**) The percentage of IFNγ^+^CD8^+^ T. (**C**) The percentage of TNFα^+^CD4^+^ T. (**D**) The percentage of TNFα^+^CD8^+^ T (*n* = 3, * *p* < 0.05; ** *p* < 0.01; *** *p* < 0.001).

FACS results showed that the proportion of T cells in the peripheral blood and spleen was significantly increased after TIL CAR-T cell treatment (Figure S5). To verify the cytotoxicity of T cells, we analyzed the expression of IFNγ and TNFα in T cells derived from the spleens of mice bearing the hCD19^+^-CT26 cells. In the PBS group, the frequency of IFNγ^+^ cells in the CD4^+^ and CD8^+^ T cells was 3.15 ± 0.54% and 9.9 ± 1.4%, respectively, much lower than that for T cell adaptive therapy. However, the frequency of IFNγ^+^CD4^+^ and IFNγ^+^CD8^+^ T cells in the CAR T and TIL CAR-T groups was significantly higher than that in the corresponding mock T and TIL-T cell groups, respectively (Figure 5A,B). CD4^+^ and CD8^+^ T cells in the spleens of the TIL CAR-T cell-treated group secreted more IFNγ than those in the CAR T group.

FACS data showed that the proportion of TNFα^+^ in CD4^+^ and CD8^+^ T cells was 40.17 ± 1.54% and 14.37 ± 1.37%, respectively, in the CAR T group. Meanwhile, the frequency of TNFα^+^ T cells was 47.8 ± 1.39% and 21.9 ± 2.04% in CD4^+^ and CD8^+^ T cells, respectively, in the TIL CAR-T group (Figure 5C,D). Significant differences were observed between the two groups. In a word, T cells in the spleens of tumor-bearing mice treated with TIL CAR-T cells showed potential cytotoxicity.

### 3.7. TIL CAR-T Cells Increased the Frequency of Memory T Cells

**Figure 6:**
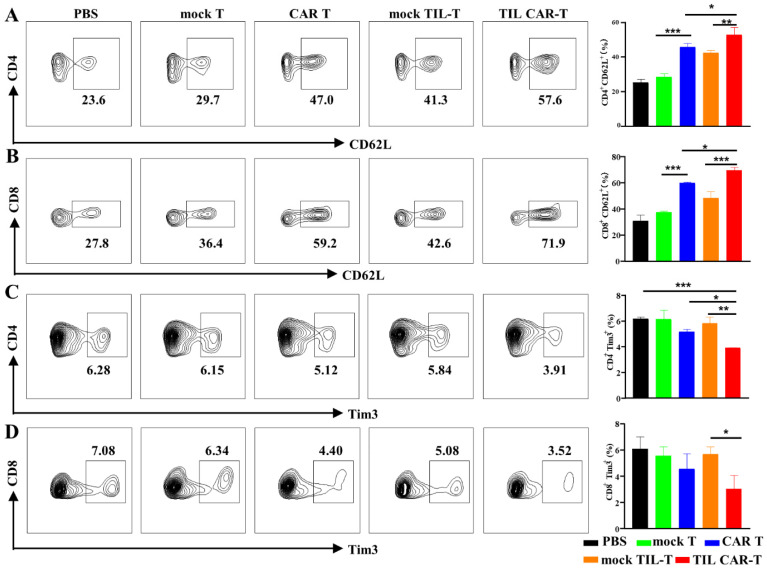
Expression of CD62L and Tim3 on CD4^+^ and CD8^+^ T cells in the spleen. The expression of CD62L and Tim3 on T cells in the spleen were detected by FACS. (**A**) The expression of CD62L on CD4^+^ T cells. (**B**) The expression of CD62L on CD8^+^ T cells. (**C**) The frequency of Tim3^+^CD4^+^ T cells. (**D**) The frequency of Tim3^+^CD8^+^ T cells (*n* = 3, * *p* < 0.05; ** *p* < 0.01; *** *p* < 0.001).

An increasing number of memory T stem cells, especially long-lived memory CD8^+^ T cells, can prevent relapse and prolong the survival time of mice. To determine the changes in memory T cells, we detected CD62L expression in splenic T cells. With CAR T administration, the frequency of CD62L^+^CD4^+^ T cells increased from 25.2 ± 1.97% to 45.67 ± 2.22%. However, the positive rate of CD62L after TIL CAR-T cell delivery was as high as 52.97 ± 4.17%. The expression of CD62L^+^ in CD8^+^ T cells was 30.77 ± 4.55% in the PBS group, 59.73 ± 0.47% in the CAR T group, and 69.43 ± 2.28% in the TIL CAR-T group (Figure 6A,B). 

### 3.8. TIL CAR-T Cells Downregulated the Expression of Tim3 in the Splenic T Cells

T cell dysfunction or exhaustion is a type of immunosuppression that hinders anti-tumor effects. Tim3 expression is a typical marker of T cell exhaustion. As shown in Figure 6C,D, the proportions of CD4^+^Tim3^+^ and CD8^+^Tim3^+^ T cells in the spleen were 3.92 ± 0.02% and 5.69 ± 0.54%, respectively, in the TIL CAR-T group, which were much smaller than those in the CAR T group. The above results indicate that TIL CAR-T cells could effectively inhibit T cell exhaustion and form immune memory compared with CAR T therapy, which could effectively exert the anti-tumor immunity of the body and prevent tumor recurrence.

### 3.9. TIL CAR-T Did Not Harm Organs In Vivo

In the T cell-treated mice, the H&E staining and histological analysis showed no significant damage to the vital organs, including the heart, liver, and kidneys (Figure S6). This proved that TIL CAR-T has a certain degree of biosafety while possessing a strong killing ability.

## 4. Discussion

In clinic, CAR T cells targeting human CD19 (hCD19) have shown remarkable efficacy against B-cell malignancies [24]. However, the therapeutic application of CAR T cells for the treatment of solid cancers has been less effective. Most CAR T studies for solid tumors yield very poor results with response rates that range from 0 to 25% [25,26,27]. Compared with hematological tumors, solid tumors pose several hurdles for this lack of efficacy, including a lack of robustly expressed tumor-exclusive antigen targets and an inefficiency of homing an intratumorally heterogeneous antigen landscape that limit treatment safety and efficacy and promote antigen escape [28,29]. Thus, to achieve sustained regression of solid tumors, CAR technology must be combined with other approaches that can simultaneously recognize neoantigens, recruit T cells into the tumor, and broaden the spectrum of T cell specificity.

The selection of suitable target antigens in solid tumors remains challenging for the therapeutic development of safe and effective CAR T-based therapies. For example, a patient’s death upon the infusion of CAR Ts was observed when target HER2, due to respiratory failure, was triggered by the low constitutive expression of HER2 in lung epithelial cells [30]. The ideal solid tumor antigen is a neoantigen that should be truly unique to tumor cells and avoid damaging normal cells. Strategies to selectively enhance T cell reactivity against oncolytic viruses using genetically defined neoantigens are currently under development. To mimic this strategy, we developed a retrovirus engineered to express the human CD19 molecules in mouse colorectal cancer cell line CT26 (hCD19^+^-CT26), which can circumvent the major challenge of identifying antigens that can be safely targeted by CD19-BBζ CAR T cells. Mock-CT26 and hCD19^+^-CT26 cells grew at the same rate after injection, indicating that the hCD19 antigen did not cause tumor rejection in the immunocompetent mice.

To investigate the function of CAR T cells in solid tumors, we transferred hCD19-reactive CAR T cells into hCD19^+^ colorectal cancer-bearing mice. We have demonstrated that hCD19 antigens can effectively prime and expand CD19-BBζ CAR T cells in vitro and in vivo. However, the therapeutic efficiency of CAR Ts can be limited due to the rapid tumor escape within the tumor, as previously reported in patients with glioblastoma treated with CAR Ts specific to EGFRvIII [10]. The results showed that expression levels of EGFRvIII decreased with an increase in the course of treatment, but the tumor was not completely cleared. Indeed, high antigen heterogeneity in solid tumors present a major mechanism of escape from CAR T cells, which typically gravitate toward a single antigen and are thus unable to recognize all tumor cells [31]. One strategy to target heterogeneous tumors more effectively is a layered approach to multi-antigen targeting.

It is worth noting that broadening tumor recognition could simultaneously increase the risk of “on-target, off-tumor” toxicity [32]. Another problem is trafficking to the tumor site. The physical barriers surrounding a solid tumor may hinder trafficking, penetration, and survival of the CAR T cells. Infiltration, accumulation, and survival of CAR T cells in solid tumors is crucial for tumor clearance [29]. So far, CAR T cells have been inadequately equipped to surmount these obstacles posed by solid tumors.

Accumulating evidence suggests that intrinsic characteristics of TIL-Ts, such as the neoantigen epitope recognized by TCR and infiltration capacity [33], may compensate for the shortcomings of CAR T cells for solid tumors. However, spontaneous TIL-T responses against tumor cells are relatively inefficient and fail to mediate tumor rejection in most cases, such as the MHC restriction, that limits the activation of them. Indeed, only a subset of patients exhibits durable responses to immune checkpoint blockades [34].

To overcome these hurdles, combining the unique advantages of CAR T and TIL-T cells is being studied to improve these therapeutic modalities in solid tumors [35]. The alternative strategy to improve the efficiency of CAR Ts and TIL-Ts is the expression of CARs in TIL-T cells. A generated CAR T derived from the TIL addresses critical hurdles to the clinical development of CAR Ts and TIL-Ts against solid tumors, as there are several potential advantages over TIL-Ts and CAR Ts. In this study, we took advantage of the cross-reactivity of the CAR Ts and TIL-Ts and developed CD19-BBζ CAR-armed TIL-T cells (TIL CAR-Ts). These cells not only could provide direct anti-tumor activity through their CAR but could also target heterogeneous tumors with a single-input receptor, which can be achieved by using TCRs of TIL-Ts that recognize a broad range of neoantigen epitopes free from MHC restriction.

We systematically compared conventional CAR T with TIL CAR-T in an immunocompetent colorectal tumor mouse model. We found that TIL CAR-Ts effectively controlled tumor growth in hCD19^+^-CT26-bearing mice with no evidence of tissue damage, indicating they did not cause “on-target, off-tumor” toxicity. Although tumors were not always eradicated, TIL CAR-Ts showed greater tumor-infiltrating capacity than CAR Ts. In particular, phenotypic analysis of splenic T cells showed significant differences in exhausted and memory T cells in mice treated with conventional CAR T or TIL CAR-T. TIL CAR-T infusion can not only effectively expand memory T cells but also inhibit exhausted T cells. They display superior anti-tumor activity when targeting tumor cells that constitutively expressed hCD19 compared with CAR Ts. This rational combination induced durable tumor regression—likely a result of pro-inflammatory cytokines induced by the TIL CAR-T support for anti-tumor T cell responses. 

At present, because many solid tumors lack tumor-specific antigens (TSAs), humanized CD19 overexpressing tumor cell lines can be used as a powerful tool and platform to evaluate the superiority of TIL CAR-Ts. Uche et al. engineered the recombinant oncolytic HSV-1 (oHSV) VC2-OVA expressing a fragment of ovalbumin (OVA) and made it a good transporter of tumor antigens and enhanced immune responses [36]. So, with the advantage of OVs, overexpression of tumor-specific antigens in the tumor microenvironment may be realized. Therefore, we plan to combine OV and TIL CAR-T technologies for the immunotherapy of solid tumors in the future, which may overcome some bottleneck problems in the treatment of solid tumors by CAR T technology. At the same time, our data indicate that both CAR and TCR are critical for eliminating antigen escape for achieving a durable regression and long-term immune memory. Given the results from checkpoint blockades and CAR T cell therapies, we hypothesized that TIL-T cells contribute to infiltration and a broad antigen target. Furthermore, TIL CAR-Ts did not completely eradicate the tumor, suggesting that the activity of TIL CAR-Ts in solid tumors should be further augmented via combination with chemotherapy, irradiation, small molecules, or other biological agents.

## 5. Conclusions

In summary, we developed a TIL CAR-T therapy that produced a better anti-tumor response than conventional CAR T cells for the control of tumor growth of colorectal cancer and prolonged the survival of tumor-bearing mice, which led us to hypothesize that CAR-armed TIL-Ts might represent an ideal platform for solid tumor therapy. Integration therapy involving CAR T cells and TIL-T cells may be an effective means of ultimately eliminating solid tumor cells. TIL CAR-Ts did not cause evident toxicity in immunocompetent mice, further encouraging their clinical application in solid tumors that are difficult to treat. Furthermore, and most importantly, CAR-armed TIL-T cells not only complement conventional CAR Ts in eliminating the primary tumor and prevent the emergence of antigen-loss variants but also form a long-term memory pool for immune surveillance.

## Data Availability

The data could be given upon reasonable request from the corresponding author.

## Supplementary Materials

The following supporting information can be downloaded at: https://www.mdpi.com/article/10.3390/cancers15235567/s1, Figure S1: Construction of hCD19^+^-CT26 and hCD19^+^-K7M2 cell line; Figure S2: TUNEL staining of CT26 cells after co-cultured with T cells (scale bar = 200 μm); Figure S3: CAR T killed hCD19-expressed K7M2 cells successfully; Figure S4: TIL CAR-T promoted immune cell infiltration in the tumor tissues; Figure S5: T cell proportion in the peripheral blood and spleen of tumor-bearing mice; Figure S6: H&E staining of kidney, heart, and lung of tumor-bearing mice.
