# Supplementary material for: TIL-Derived CAR T Cells Improve Immune Cell Infiltration and Survival in the Treatment of CD19-Humanized Mouse Colorectal Cancer
Source: Cancers (Basel). 2023 Nov 24;15(23):5567. doi: 10.3390/cancers15235567 (PMC10705672; doi:10.3390/cancers15235567)
Supplement: Supplementary file 1 [file cancers-15-05567-s001.zip › cancers-2644150-supplementary.pdf]

Supplementary Materials

TIL-Derived CAR T Cells Improve Immune Cell Infiltration and Survival in the Treatment of CD19-Humanized Mouse Colorectal Cancer

Can Zhu [1,2,†], Yuanyuan Zhao [2,3,†], Jiaheng He [4], Huan Zhao [1,2], Li Ni [1,2], Xinyi Cheng [1], Yida Chen [1], Liqian Mu [5], Xiaojun Zhou [5], Qin Shi [1,2,*] and Jie Sun [1,2,*]

1 Department of Orthopedics, The First Affiliated Hospital of Soochow University, Orthopedic Institute of Soochow University, Suzhou Medical College, Soochow University, 899 Pinghai Road, Suzhou 215031, China; 20224032012@stu.suda.edu.cn (C.Z.); zhaohuan@suda.edu.cn (H.Z.); nili@suda.edu.cn (L.N.); 20224232047@stu.suda.edu.cn (X.C.); 20224232049@stu.suda.edu.cn (Y.C.)
2 National Clinical Research Center for Hematologic Diseases, The First Affiliated Hospital of Soochow University, 899 Pinghai Road, Suzhou 215031, China; zyy@suda.edu.cn
3 Department of Pathology, School of Biology and Basic Medical Sciences, Suzhou Medical College, Soochow University, Suzhou 215123, China
4 Department of Orthopaedics, The Affiliated Jiangsu Shengze Hospital of Nanjing Medical University, No. 1399, Market West Road, Shengze Town, Suzhou 215000, China; 18896719637@163.com
5 Department of General Surgery, The First Affiliated Hospital of Soochow University, Suzhou Medical College, Soochow University, 899 Pinghai Road, Suzhou 215031, China; muliqian@suda.edu.cn (L.M.); chowxj@126.com (X.Z.)
* Correspondence: shiqin@suda.edu.cn (Q.S.); sunjie0829@suda.edu.cn (J.S.)
† These authors contributed equally to this work.

Supplementary Figure S1 to S6

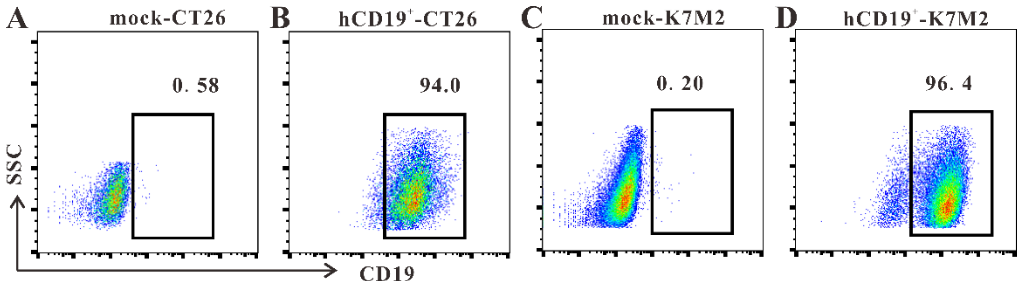

***Figure S1*. Construction of hCD19^{+}-CT26 and hCD19^{+}-K7M2 cell line.**

CD19 expression on the tumor cells were detected by FACS. (A) mock-CT26 cells infected with MSCV-GFP control retrovirus; (B) MSCV-hCD19 retrovirus infected CT26 cells (hCD19^{+}-CT26) by sorting; (C) mock-K7M2 cells infected with MSCV-GFP control retrovirus; (D) MSCV-hCD19 retrovirus infected K7M2 (hCD19^{+}-K7M2) cells by sorting.

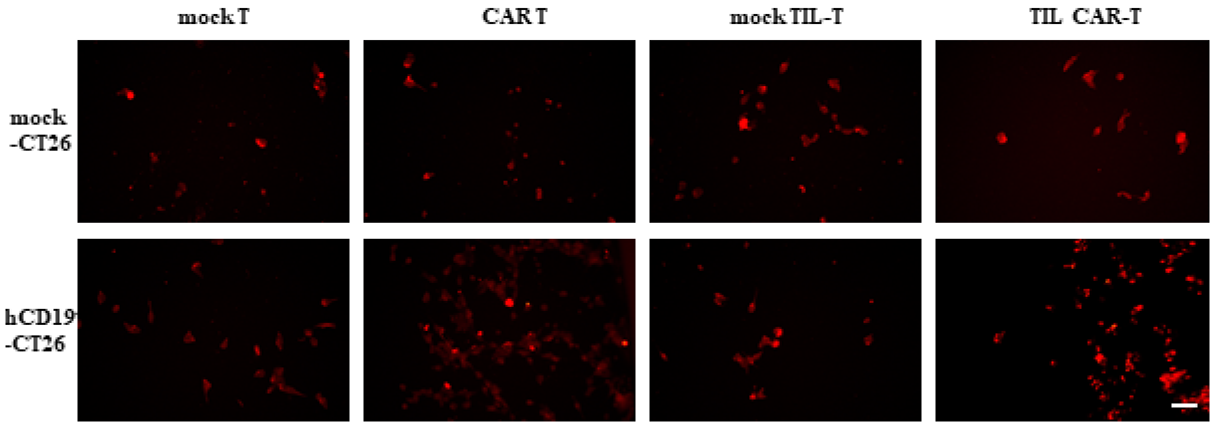

***Figure S2.* TUNEL-staining of CT26 cells after co-cultured with T cells. (scale bar= 200 μm)**

Both CD19-BBζ CAR T and TIL CAR-T could effectively kill the hCD19^{+}-CT26 cells and lead to apoptosis of the tumor cells.

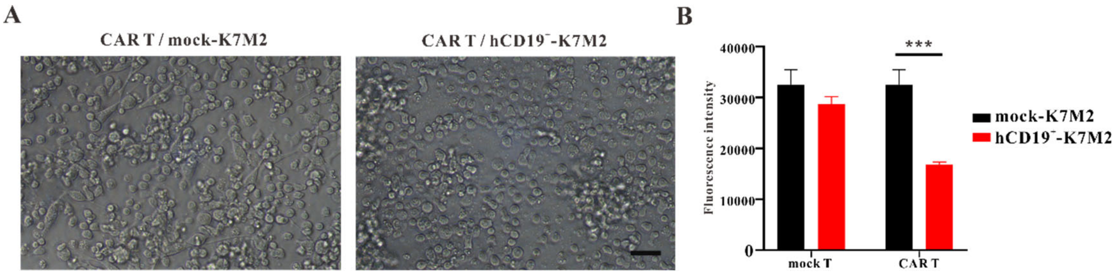

Figure S3. **CAR T killed hCD19-expressed K7M2 cells successfully.**

The CAR T cells were co-cultured with hCD19$^+$-K7M2 cells and mock-K7M2 cells for 12 h.(A) Representative images of CAR T co-cultured with K7M2 cells (scale bar = 200 μm); (B) fluorescence intensity of K7M2 cells detected by Resazurin dye solution (n = 5, $^{***}P < 0.001$).

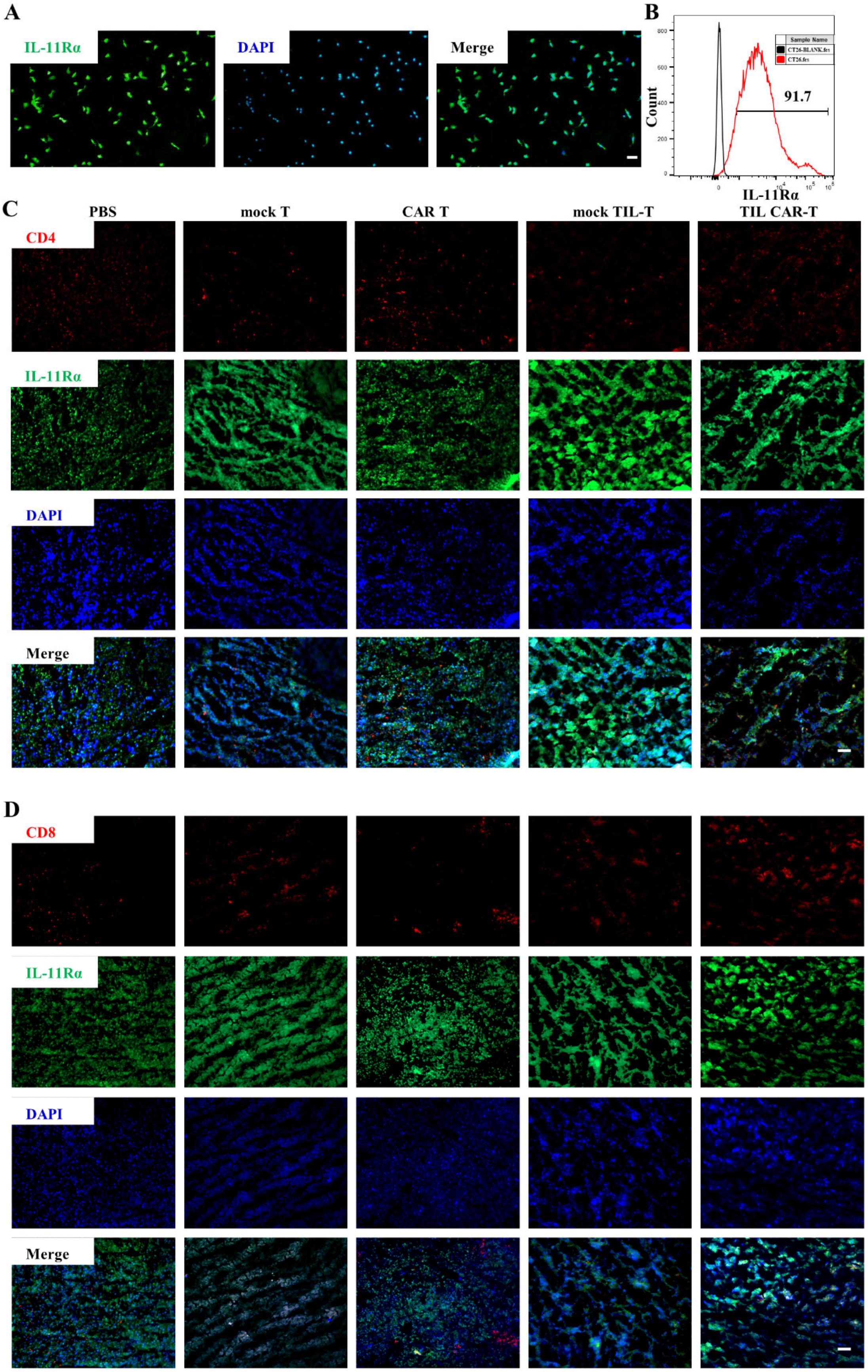

Figure S4. TIL CAR-T promoted immune cell infiltration in the tumor tissues. IL-

11Rα is highly expressed in Colorectal cancer[1,2] and considered to be a tumor-associated antigen (TAA) for a target in cancer therapy[3,4]. (A) Representative images of IL-11Rα (green) and DAPI (blue) fluorescence staining (scale bar = 200 μm) ;(B) The expression of IL-11Rα on CT26 cells by FACS; (C) Representative images of CD4 (red), IL-11Rα(green) and DAPI (blue) fluorescence staining (scale bar =50 μm); (D) Representative images of CD4 (red), IL-11Rα(green) and DAPI (blue) fluorescence staining (scale bar = 50 μm).

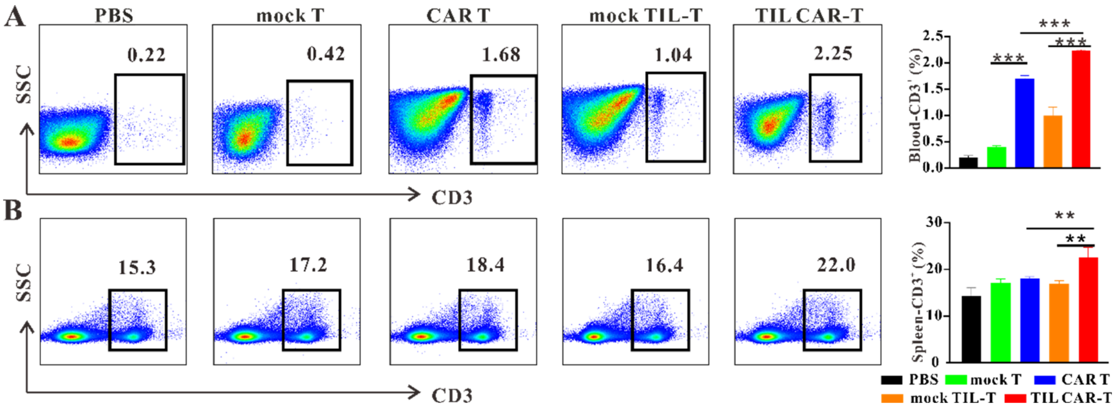

***Figure S5.* T cell proportion in the peripheral blood and spleen of tumor-bearing mice.**

The frequency of T cell in the blood and spleen were detected by FACS. (A) The percentage of T cell in peripheral blood; (B) The percentage of T cells in spleen of tumor-bearing mice after cell transfusion (n = 3, $^{**}P < 0.01$; $^{***}P < 0.001$).

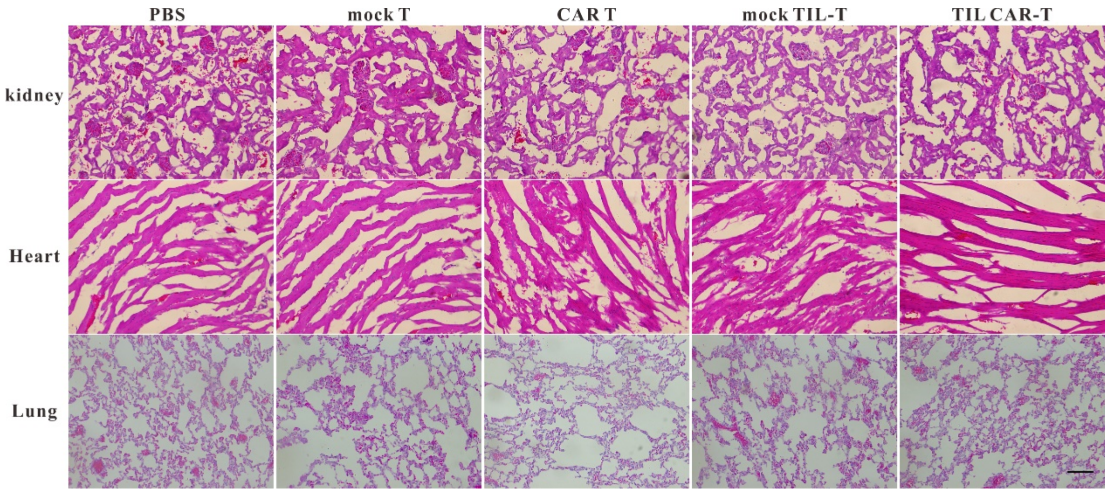

Figure S6. **H&E staining of kidney, heart, and lung of tumor-bearing mice.**

H&E staining was performed on the kidney, heart, and lung sections of different groups of tumor-bearing mice, and no obvious pathological injury was observed (scale bar = 200 μm).

References

1. Yamazumi, K.; Nakayama, T.; Kusaba, T.; Wen, C.Y.; Yoshizaki, A.; Yakata, Y.; Nagayasu, T.; Sekine, I. Expression of interleukin-11 and interleukin-11 receptor alpha in human colorectal adenocarcinoma; immunohistochemical analyses and correlation with clinicopathological factors. *World J Gastroenterol* **2006**, *12*, 317-321, doi:10.3748/wjg.v12.i2.317.

2. Yoshizaki, A.; Nakayama, T.; Yamazumi, K.; Yakata, Y.; Taba, M.; Sekine, I. Expression of interleukin (IL)-11 and IL-11 receptor in human colorectal adenocarcinoma: IL-11 up-regulation of the invasive and proliferative activity of human colorectal carcinoma cells. *Int J Oncol* **2006**, *29*, 869-876.

3. Cardó-Vila, M.; Marchiò, S.; Sato, M.; Staquicini, F.I.; Smith, T.L.; Bronk, J.K.; Yin, G.; Zurita, A.J.; Sun, M.; Behrens, C.; et al. Interleukin-11 Receptor Is a Candidate Target for Ligand-Directed Therapy in Lung Cancer: Analysis of Clinical Samples and BMTP-11 Preclinical Activity. *Am J Pathol* **2016**, *186*, 2162-2170, doi:10.1016/j.ajpath.2016.04.013.

4. Wu, Q.; He, Y.; Gu, C.; Jiang, J.; Zhou, H.; Zhou, S. Binding Specificity of Radiolabeled Cyclic Peptide 153Sm-DTPA-c(CGRRAGGSC) to MHCC97-H Human Liver Cancer Cells and its Antitumor Effects in vivo. *Technol Cancer Res Treat* **2016**, *15*, Np1-np9, doi:10.1177/1533034615604785.
